# Systemic Administration of a Site-Targeted Complement Inhibitor Attenuates Chronic Stress-Induced Social Behavior Deficits and Neuroinflammation in Mice

**DOI:** 10.3390/cells13231988

**Published:** 2024-12-02

**Authors:** Amit Kumar Madeshiya, Brandi Quintanilla, Carl Whitehead, Stephen Tomlinson, Anilkumar Pillai

**Affiliations:** 1 Translational Psychiatry Program, Faillace Department of Psychiatry and Behavioral Sciences, The University of Texas Health Science Center at Houston (UTHealth), Houston, TX 77054, USA; amit.kgmu21@gmail.com (A.K.M.); cwhitehead1@augusta.edu (C.W.); 2 Department of Pharmacology and Immunology, Medical University of South Carolina, Charleston, SC 29425, USA; 3 Ralph Johnson VA Medical Center, Charleston, SC 29401, USA; 4 Research and Development, Charlie Norwood VA Medical Center, Augusta, GA 30904, USA

**Keywords:** complement, C2-Crry, social behavior, inflammation

## Abstract

Chronic stress, a risk factor for many neuropsychiatric conditions, causes dysregulation in the immune system in both humans and animal models. Additionally, inflammation and synapse loss have been associated with deficits in social behavior. The complement system, a key player of innate immunity, has been linked to social behavior impairments caused by chronic stress. However, it is not known whether complement inhibition can help prevent neuroinflammation and behavioral deficits caused by chronic stress. In this study, we investigated the potential of a site-targeted complement inhibitor to ameliorate chronic stress-induced changes in social behavior and inflammatory markers in the prefrontal cortex (PFC) and hippocampus. Specifically, we investigated the use of C2-Crry, which comprises a natural antibody-derived single-chain antibody (ScFv) targeting domain-designated C2, linked to Crry, a C3 activation inhibitor. The C2 targeting domain recognizes danger-associated molecular patterns consisting of a subset of phospholipids that become exposed following cell stress or injury. We found that systemic administration of C2-Crry attenuated chronic stress-induced social behavioral impairments in mice. Furthermore, C2-Crry administration significantly decreased microglia/macrophage and astrocyte activation markers in the PFC and hippocampus. These findings suggest that site-targeted complement inhibition could offer a promising, safe, and effective strategy for treating chronic stress induced behavioral and immune function disorders.

## 1. Introduction

Chronic stress is a risk factor for many psychiatric disorders [1,2]. Chronic stress is associated with immune dysregulation in both animal models and humans [3]. Immune activation is causally linked to the development of behavioral deficits [4]. Systemic inflammation has been shown to result in impairments in behavior in humans [5,6,7,8]. In addition, inflammatory cytokines are well-known contributors to the pathophysiology of depression and other chronic stress conditions [9]. A meta-analysis examining cytokine levels in individuals with major depression found that depressed subjects had significantly elevated serum levels of TNF-α and IL-6 compared to non-depressed controls [10]. Furthermore, studies have demonstrated that chronic stress exposure leads to elevated levels of TNF-α, IL-1β, and IL-6 in the hippocampus of rats [11]. Similarly, cytokine administration induces increased neuroinflammation and behavior deficits in rodents [12]. A number of different chronic stress paradigms, including chronic unpredictable stress [13,14], chronic adolescent stress [15], and persistent social defeat [16] promote abnormalities in behavior, including behavioral despair, anxiety-like behavior, anhedonia, and cognitive deficits and social behavior impairments in animals. Conversely, inhibition of immune activation attenuates stress-induced behavioral deficits [17,18,19]. These results support the connection between immune pathways and behavioral alterations.

Several recent studies have explored the potential of novel therapies aimed at inflammatory pathways in chronic stress-induced behavior deficits. For example, ginsenoside Rb1 (GRb1) reversed depressive-like behaviors induced by chronic stress [20]. Treatment with GRb1 also reduced the number of astrocytes and microglia and suppressed the inflammatory signaling pathway in the astrocytes of the hippocampus [20]. Another study by Jiang et al. found that Asperosaponin VI improved depression-like behaviors in mice exposed to chronic mild stress via a shift of hippocampal microglia from a pro-inflammatory phenotype to a neuroprotective phenotype [21]. Pioglitazone, a peroxisome proliferator-activated receptor γ agonist, has been shown to promote a neuroprotective phenotype in microglia and alleviates depression-like behaviors in chronic mild stress-treated mice [22]. Administration of salvianolic acid B significantly decreased the expression of pro-inflammatory cytokines IL-1β and TNF-α, and markedly increased the expression of anti-inflammatory cytokines IL-10 and TGF-β in the hippocampus and cortex of chronic mild stress-treated mice [23]. Although the above studies are interesting, the molecular mechanism underlying chronic stress-induced neuroinflammation and behavior deficits are still not well understood.

The complement system is a crucial element of the innate immune system and plays an important role in synaptic plasticity [24]. The complement system is principally activated via three different pathways: the classical, alternative, and lectin pathways. The classical pathway is triggered by the C1 protein complex, which consists of C1q, C1r, and C1s. Once activated, the C1 complex facilitates the cleavage of C3 into C3a and C3b. The lectin pathway produces the same C3 products after activation via mannose-binding lectin (MBL). The alternative pathway is spontaneously activated to generate C3 cleavage products, and also serves to amplify the classical and lectin pathways. Thus, C3 is the hub for all complement activation pathways and subsequently results in the downstream generation of biologically active C5a and the membrane attack complex. The complement inhibitor used in the current study functions at this central C3 activation step. Elevated levels of C3a and C5a have been observed in patients with bipolar disorder [25]. Additionally, patients with major depressive disorder (MDD) exhibited higher levels of C5 in their cerebrospinal fluid compared to healthy controls [26]. Likewise, our previous study revealed elevated levels of C3 mRNA in the prefrontal cortex (PFC) of individuals who died by suicide and were depressed [14]. Furthermore, we discovered that blocking C3a signaling in C3a receptor knockout mice reduces depressive-like behavior induced by chronic stress [14]. Although these findings highlight a vital role of the complement pathway in prolonged stress-induced changes in behavior and immune response, whether inhibition of activated complement has any significant effect on attenuating chronic stress-diced neuroinflammation and behavior deficits remains unclear.

Since complement components are essential for immune defense against infections [27], tissue repair and regeneration [28], and other homeostatic mechanisms, systemic inhibition is likely to result in undesirable off-target effects. A recently described approach for localizing complement inhibition specifically to sites of complement activation utilized a targeting vehicle that recognizes danger-associated molecular patterns (DAMPs) expressed on the cell surface. In this approach, a single-chain antibody (scFv) derived from a natural self-reactive IgM antibody (designated C2) was linked to murine Crry that blocks all pathways at the C3 activation stage [29]. The resulting fusion protein, C2-Crry, identifies a specific group of phospholipids in both rodents and humans [30]. These phospholipids represent cryptic neoepitopes expressed on stressed and injured cells, but not on healthy cells [30]. C2-Crry has been shown to be protective in mouse models of lung injury [29], arthritis [31], and hepatic ischemia [32]. CR2-mediated targeting of a complement activation inhibitor has been shown to enhance local bioavailability and maintain host infection resistance in mouse models of intestinal ischemic injury [33,34]. Therefore, as compared to other complement-based inhibitors, a target-specific complement inhibition using C2-Crry is beneficial and avoids undesirable side effects. Since chronic stress exposure can have significant systemic effects on the body, we tested the potential of systemic administration of C2-Crry on chronic stress-induced neuroinflammation and social behavior deficits in the present study.

We found that C2-Crry treatment reduced social behavior deficits in mice caused by chronic stress. In addition, administration of C2-Crry significantly reduced chronic stress-induced increases in the proinflammatory and astrocyte activation markers in the PFC and hippocampus, key brain regions associated in social behavior. Together, these findings suggest that targeted complement inhibitors could offer a new, effective, and safe method for treating chronic stress-induced abnormalities in behavior and immune function.

## 2. Materials and Methods

**Mice:** C57BL/J (Stock no. 000664) male mice (6–8 weeks old) were purchased from The Jackson Laboratory (Bar Harbor, ME, USA). Mice were group-housed (five mice per cage) in individually ventilated cages and maintained on a 12 h light–dark cycle in the institutional animal housing facility. Mice had ad libitum access to food and water. Experimental procedures, including stress paradigm and behavior tests, were conducted during the light phase, adhering to a pre-approved protocol from the Institutional Animal Care and Use Committee (IACUC) of the University of Texas Health Science Center at Houston, ensuring ethical and regulatory compliance.

**Stress paradigm:** To study the chronic-stress-induced social behavior deficit, the animals were subjected to the chronic unpredictable stress (CUS) paradigm as described in our previous studies [14,35]. Briefly, prior to starting the CUS protocol, all the animals were singly housed, keeping other housing conditions the same as mentioned above. All the animals were exposed to 1–3 different stressors like confinement, forced swimming, paired housing, inversion of the light/dark cycle, empty water bottles, and food restrictions for 21 days during morning, mid-day, and/or evening in an unpredictable manner. The details of schedule, duration, and type of stressor are given in Table S1.

**Figure 1:**
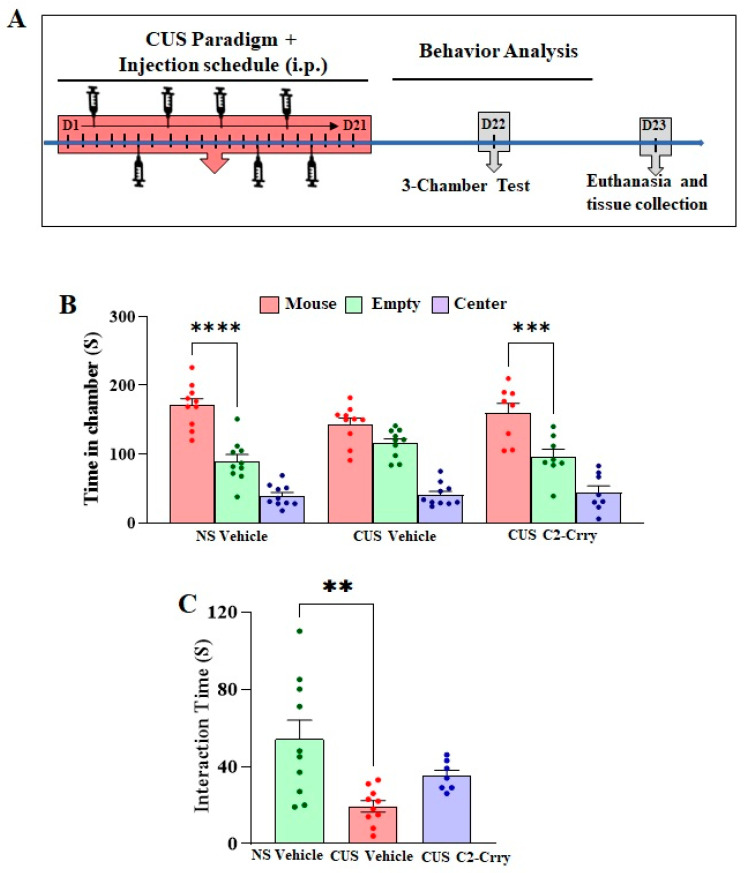
Systemic administration of C2-Crry attenuates chronic stress-induced social behavior deficits in three-chamber test. (**A**) Experimental design showing the schedule of chronic unpredictable stress and intraperitoneal (i.p.) C2-Crry/Vehicle administration in mice followed by the social behavior test and tissue collection. (**B**) Three-chamber test: Time in the chamber (S): two-way ANOVA (F (4, 75) = 2.481; *p* = 0.0509) treatment X stress interaction followed by Tukey’s multiple comparison post hoc test, **** *p* < 0.0001 (NS vehicle: Mouse chamber vs. NS vehicle: Empty chamber); *p* = 0.3613 (CUS vehicle: Mouse chamber vs. CUS vehicle CUS: Empty chamber); *** *p* = 0.0005 (CUS C2-Crry: Mouse chamber vs. CUS C2-Crry CUS: Empty chamber), n = 8–10 per group. (**C**) Reciprocal social interaction test: one-way ANOVA (F (2, 24) = 7.483; *p* = 0.0030) followed by Tukey’s multiple comparison post hoc test, ** *p* = 0.0021 (NS vehicle vs. CUS vehicle) and *p* = 0.2707 (CUS vehicle vs. CUS C2-Crry), n = 7–10 per group.

**Drug treatment:** To investigate the effect of inhibiting C3 activation, mice were administered C2-Crry (10 mg/kg) or vehicle (saline) intraperitoneally (i.p.) 1 h before stress exposure during the CUS paradigm. Mice were given three (one/day) injections at the beginning of the stress paradigm followed by twice/week injections (Figure 1A). C2-Crry was constructed, expressed in Expi293 cells, and purified as previously described [29]. Purified C2-Crry gave a single band by SDS-PAGE of appropriate molecular mass. Protein concentration was determined by BCA protein assay, and a 10 mg/Kg dose was utilized based on a previous dose response study showing that this dose provides maximal protection in a mouse model of complement-dependent injury [33].

**Social behavior test:** The test was conducted using a three-chamber apparatus constructed from waterproof and sanitized Plexiglas (26 cm × 45 cm × 23 cm). Each dividing partition of the chambers features openings on each wall to allow free access to the other chambers. Two identical wire containers (9 cm in diameter) were vertically placed in the side chambers of the apparatus. These wire containers were designed to allow for air exchange only, preventing any physical contact between the test mouse and the stranger mouse. All animals were acclimatized in the behavior room for at least one hour prior to testing. During the initial 5 min, the mouse was permitted to move freely in the apparatus to habituate. After this habituation period, an age-, sex-, and background-matched stranger mouse was placed in one of the wire containers, referred to as the mouse chamber, while the other container remained empty, designated as the empty chamber. The time spent by the test mouse in each chamber (stranger mouse chamber, empty chamber, and center chamber) was recorded on video. Social behavior was assessed by scoring the amount of time spent in the mouse chamber compared to the empty chamber during the 5-min testing period. Mice without social behavior deficits tend to spend more time in a stranger mouse chamber compared to the empty chamber, while the mice with social behavior deficits will not have a visit preference between both chambers.

**Reciprocal social interaction test:** In this test, the stranger mouse was permitted to move freely throughout the entire three-chamber apparatus alongside the test mouse, allowing for physical interaction. The interactions between the mice were characterized by close physical contact, nose-to-nose sniffing, anogenital sniffing, and grooming. The duration of interactions (initiated solely by the test mouse) was recorded on video for 5 min. All behavioral tests took place in a designated behavior room equipped with consistent ambient lighting, temperature, background sound, and pressure. All recordings from the behavioral experiments were scored without knowledge of the treatment conditions.

**Quantitative mRNA expression analysis:** Total RNA was extracted from the prefrontal cortex (PFC) and hippocampus using a commercially available total RNA Isolation kit (SV RNA Isolation, Promega, Madison, WI, USA), following the manufacturer’s instructions. The concentration of the extracted RNA was quantified using a nanodrop 8000 spectrophotometer (Thermo Scientific 8000; Waltham, MA, USA). Next, complementary DNA (cDNA) was synthesized using the iScript^TM^ cDNA Synthesis Kit (BioRad, Hercules, CA, USA) according to the manufacturer’s protocol. The analysis of mRNA expression for macrophage/microglia M1/M2 and astrocyte A1/A2 markers was conducted using iTaqTM Universal SYBR^®^ Green Supermix (Bio-Rad). Primers were obtained from Integrated DNA Technologies. All qRT-PCR reactions were performed using a MasterCycler (Quant Studio-7 Real-Time PCR Systems, Thermo Fisher Scientific, USA). For quantification, the Ct values of the target genes were normalized to the expression level of the housekeeping gene beta2-microglobulin (B2M), and the fold change was calculated. A list of the primers used is provided in Table S2.

**Statistical Analysis:** The data were presented as mean ± SEM. An Unpaired Student’s *t*-test was used for two-group comparisons, while analysis of variance (ANOVA) was employed for multiple-group comparisons. Post hoc analyses were conducted using Tukey’s test. Grubbs’ test was applied to identify significant outliers using an online GraphPad application (https://www.graphpad.com/quickcalcs/grubbs1/, accessed on 11 April 2024). GraphPad Prism (Version 9.0.0) was used for all statistical analyses and graph preparation. A significance level of *p* < 0.05 was considered statistically significant.

## 3. Results

### 3.1. Systemic Administration of C2-Crry Attenuates Chronic Stress-Induced Social Behavior Deficits

To assess the impact of the C2-Crry fusion protein on social behavior deficits induced by chronic stress, mice received three injections (one per day) at the start of the stress paradigm, followed by biweekly injections. Social behavior tests were conducted 24 h after the final stressor (Figure 1A). In the three-chamber test, vehicle-injected normal control (NS) mice spent more time in the chamber containing the stranger mouse compared to the empty cage chamber (Figure 1B). In contrast, vehicle-injected chronic unpredictable stress (CUS) mice displayed no preference for either chamber. However, CUS mice treated with C2-Crry spent more time in the chamber with the stranger mouse than in the empty cage chamber (Figure 1B). Additionally, in the reciprocal social interaction test, vehicle-injected CUS mice exhibited reduced interaction with the stranger mouse compared to vehicle-injected NS mice (Figure 1C). In contrast, treatment with C2-Crry resulted in a non-significant attenuation in the reduction in interaction time caused by CUS in the mice (Figure 1C). These findings indicate that complement inhibition using C2-Crry effectively alleviates social behavior deficits induced by chronic stress in mice.

### 3.2. C2-Crry Treatment Attenuates Chronic Stress-Induced Increases in Microglia/Macrophage Activation Markers in the PFC and Hippocampus

**Figure 2:**
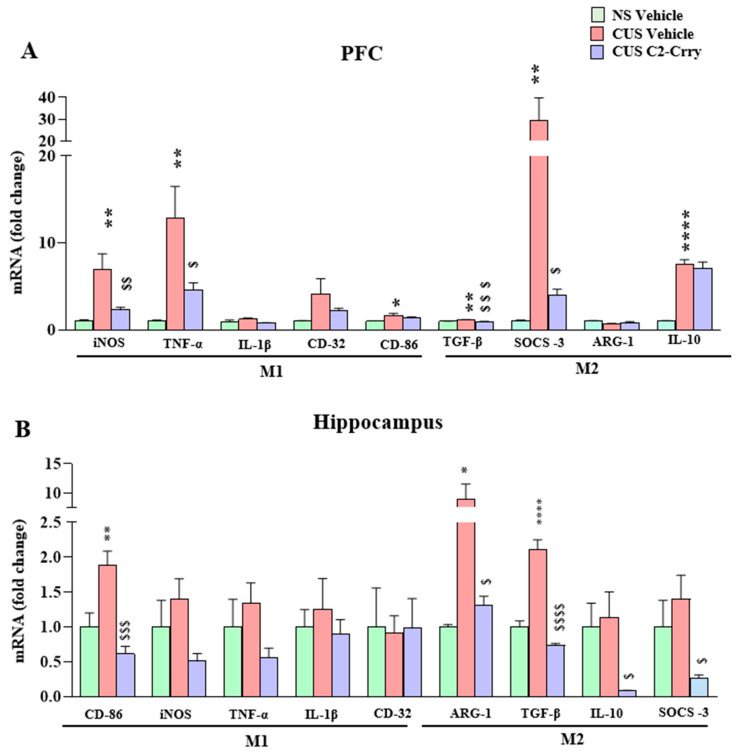
Systemic administration of C2-Crry attenuates chronic stress-induced increases in the mRNA levels of M1/M2 markers iNOS, TNF-α, TGF-β, and SOCS-3 in the PFC, and CD-86 and ARG-1 in the hippocampus. (**A**) *mRNA expression (fold change) of the microglia/macrophage M1 phenotype markers in the PFC:* iNOS, one-way ANOVA (F (2, 21) = 9.611; *p* = 0.0011), 1.0 ± 0.17 vs. 7.0 ± 1.7, ** *p* = 0.0012 (NS vehicle vs. CUS vehicle) and 7.0 ± 1.7 vs. 2.3 ± 0.33, ^$$^ *p* = 0.0097 (CUS vehicle vs. CUS C2-Crry), n = 8 per group. TNF-α, one-way ANOVA (F (2, 21) = 8.213; *p* = 0.0023), 1.0 ± 0.13 vs. 13.0 ± 3.6, ** *p* = 0.0020 (NS vehicle vs. CUS vehicle) and 13.0 ± 3.6 vs. 4.6 ± 0.79, ^$^ *p* = 0.0309 (CUS vehicle vs. CUS C2-Crry), n = 8 per group. IL-1β, one-way ANOVA (F (2, 21) = 3.161; *p* = 0.0631), 1.0 ± 0.13 vs. 1.2 ± 0.16, *p* = 0.3839 (NS vehicle vs. CUS vehicle) and 1.2 ± 0.16 vs. 0.80 ± 0.04, *p* = 0.0509 (CUS vehicle vs. CUS C2-Crry), n = 8 per group. CD-32, one-way ANOVA (F (2, 21) = 2.583; *p* = 0.0993), 1.0 ± 0.09 vs. 4.2 ± 1.7, *p* = 0.0858 (NS vehicle vs. CUS vehicle) and 4.2 ± 1.7 vs. 2.2 ± 0.30, *p* = 0.3568 (CUS vehicle vs. CUS C2-Crry), n = 8 per group. CD-86, one-way ANOVA (F (2, 20) = 4.821; *p* = 0.0196), 1.0 ± 0.05 vs. 1.7 ± 0.22, * *p* = 0.0148 (NS vehicle vs. CUS vehicle) and 1.7 ± 0.22 vs. 1.4 ± 0.13, *p* = 0.2820 (CUS vehicle vs. CUS C2-Crry), n = 7–8 per group. *mRNA expression (fold change) of the microglia/macrophage M2 phenotype markers in the PFC:* TGF-β, one-way ANOVA (F (2, 21) = 10.39; *p* = 0.0007), 1.0 ± 0.02 vs. 1.2 ± 0.04, ** *p* = 0.0076 (NS vehicle vs. CUS vehicle) and 1.2 ± 0.04 vs. 0.96 ± 0.02, ^$$$^ *p* = 0.0008 (CUS vehicle vs. CUS C2-Crry), n = 8 per group. SOCS-3, one-way ANOVA (F (2, 21) = 6.822; *p* = 0.0052), 1.0 ± 0.12 vs. 29.44 ± 10.34, ** *p* = 0.0080 (NS vehicle vs. CUS vehicle) and 29.44 ± 10.34 vs. 3.95 ± 0.74, ^$^ *p* = 0.0176 (CUS vehicle vs. CUS C2-Crry), n = 8 per group. ARG-1, one-way ANOVA (F (2, 11) = 3.132; *p* = 0.0838), 1.0 ± 0.09 vs. 0.70 ± 0.05, *p* = 0.0702 (NS vehicle vs. CUS vehicle) and 0.70 ± 0.05 vs. 0.79 ± 0.16, *p* = 0.7563 (CUS vehicle vs. CUS C2-Crry), n = 3–7 per group. IL-10, one-way ANOVA (F (2, 21) = 54.61; *p* < 0.0001), 1.0 ± 0.07 vs. 7.6 ± 0.46, **** *p* < 0.0001 (NS vehicle vs. CUS vehicle) and 7.6 ± 0.46 vs. 7.1 ± 0.72, *p* = 0.7510 (CUS vehicle vs. CUS C2-Crry), n = 8 per group. (**B**) *mRNA expression (fold change) of the microglia/macrophage M1 phenotype markers in the hippocampus:* CD-86, one-way ANOVA (F (2, 20) = 13.41; *p* = 0.0002), 1.0 ± 0.20 vs. 1.9 ± 0.20, ** *p* = 0.0055 (NS vehicle vs. CUS vehicle) and 1.9 ± 0.20 vs. 0.62 ± 0.11, ^$$$^ *p* = 0.0002 (CUS vehicle vs. CUS C2-Crry), n = 7–8 per group. iNOS, one-way ANOVA (F (2, 21) = 2.454; *p* = 0.1102), 1.0 ± 0.38 vs. 1.4 ± 0.29, *p* = 0.5915 (NS vehicle vs. CUS vehicle) and 1.4 ± 0.29 vs. 0.51 ± 0.11, *p* = 0.0924 (CUS vehicle vs. CUS C2-Crry), n = 8 per group. TNF-α, one-way ANOVA (F (2, 21) = 1.752; *p* = 0.1978), 1.0 ± 0.40 vs. 1.3 ± 0.29, *p* = 0.6952 (NS vehicle vs. CUS vehicle) and 1.3 ± 0.29 vs. 0.56 ± 0.13, *p* = 0.1729 (CUS vehicle vs. CUS C2-Crry), n = 8 per group. IL-1β, one-way ANOVA (F (2, 21) = 0.3544; *p* = 0.7057), 1.0 ± 0.25 vs. 1.3 ± 0.44, *p* = 0.8324 (NS vehicle vs. CUS vehicle) and 1.3 ± 0.44 vs. 0.90 ± 0.21, *p* = 0.6959 (CUS vehicle vs. CUS C2-Crry), n = 8 per group. CD-32, one-way ANOVA (F (2, 21) = 0.01182; *p* = 0.9883), 1.0 ± 0.56 vs. 0.91 ± 0.25, *p* = 0.9883 (NS vehicle vs. CUS vehicle) and 0.91 ± 0.25 vs. 0.98 ± 0.43, *p* = 0.9927 (CUS vehicle vs. CUS C2-Crry), n = 8 per group. *mRNA expression (fold change) of the microglia/macrophage M2 phenotype markers in the hippocampus:* ARG-1, one-way ANOVA (F (2, 11) = 7.596; *p* = 0.0085), 1.0 ± 0.03 vs. 8.9 ± 2.6, * *p* = 0.0175 (NS vehicle vs. CUS vehicle) and 8.9 ± 2.6 vs. 1.3 ± 0.13, ^$^ *p* = 0.0158 (CUS vehicle vs. CUS C2-Crry), n = 4–5 per group. TGF-β, one-way ANOVA (F (2, 18) = 64.35; *p* < 0.0001), 1.0 ± 0.09 vs. 2.1 ± 0.14, **** *p* < 0.0001 (NS vehicle vs. CUS vehicle) and 2.1 ± 0.14 vs. 0.74 ± 0.02, ^$$$$^ *p* < 0.0001 (CUS vehicle vs. CUS C2-Crry), n = 6–8 per group. IL-10, one-way ANOVA (F (2, 18) = 4.860; *p* = 0.0205), 1.0 ± 0.34 vs. 1.1 ± 0.37, *p* = 0.9355 (NS vehicle vs. CUS vehicle) and 1.1 ± 0.37 vs. 0.08 ± 0.01, ^$^ *p* = 0.0322 (CUS vehicle vs. CUS C2-Crry), n = 6–8 per group. SOCS-3, one-way ANOVA (F (2, 21) = 3.726; *p* = 0.0412), 1.0 ± 0.38 vs. 1.4 ± 0.34, *p* = 0.6145 (NS vehicle vs. CUS vehicle) and 1.4 ± 0.34 vs. 0.27 ± 0.04, ^$^ *p* = 0.0350 (CUS vehicle vs. CUS C2-Crry), n = 8 per group. Tukey’s multiple comparison post hoc test was used in the analysis. * *p* < 0.05; ** *p* < 0.01 and **** *p* < 0.00001 represent the comparison between NS Vehicle vs CUS Vehicle. ^$^ *p* < 0.05; ^$$^ *p* < 0.01; ^$$$^ *p* < 0.001 and ^$$$$^ *p* < 0.00001 represent the comparison between CUS Vehicle vs CUS C2-Crry.

There is growing evidence that abnormal neuroinflammatory responses such as higher concentrations of pro-inflammatory cytokines such as TNFα and IL-1β in the brain promote susceptibility to psychiatric conditions like depression [9,10,36,37,38,39]. In addition, chronic stress exposure induces both microglia/macrophage activation and pro-inflammatory cytokines in the brains of animal models [40]. It is known that microglia/macrophages transition between a pro-inflammatory (M1-like) and an anti-inflammatory (M2-like) phenotype in response to disturbance of tissue homeostasis [41,42,43]. Our earlier study found an increase in the levels of M1-like markers in the PFC of mice following CUS exposure [44]. Here, we determined the mRNA levels of markers associated with an M1-like phenotype (iNOS, TNFα, IL1b, CD32 and CD86) and an M2-like phenotype (TGFb, IL-10, suppressor of cytokine signaling 3 (Socs3), and arginase-1 (Arg1)) in the PFC and hippocampus. qPCR results showed significant increases in the expression levels of iNOS, TNFα, CD-86, IL-10, TGFb, and Socs3 in the PFC of CUS-exposed mice (Figure 2A). Notably, C2-Crry treatment ameliorated the increases in iNOS, TNFα, TGFb, and Socs3 levels in the PFC of CUS mice (Figure 2A). In the hippocampus, we observed increases in CD-86, TGFb, and Arg1 following CUS, which were attenuated by C2-Crry treatment (Figure 2B). In addition, we found that C2-Crry treatment in CUS-exposed mice resulted in significant decreases in IL-10 and Socs3 mRNA levels in the hippocampus (Figure 2B). We did not find any significant change in IL1b and CD32 levels by stress or C2-Crry treatment. Together, C2-Crry significantly attenuated chronic stress-induced changes in M1/M2 markers in the PFC and hippocampus compared to controls, suggesting a modulatory effect on stress-induced microglial activation.

### 3.3. C2-Crry Treatment Attenuates Chronic Stress-Induced Increases in Astrocyte Activation Markers in the PFC and Hippocampus

**Figure 3:**
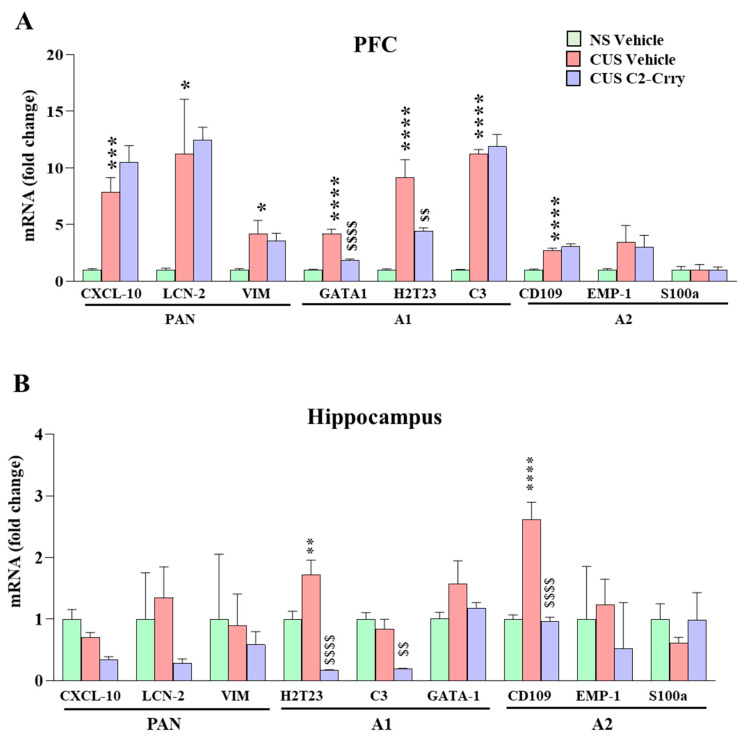
Systemic administration of C2-Crry attenuates chronic stress-induced increases in the mRNA levels of A1/A2 markers GATA-1, H2T23 in the PFC, and H2T23 and CD = 109 in hippocampus. (**A**) *mRNA expression (fold change) of the astrocyte activation PAN markers in the PFC:* CXCL-10, one-way ANOVA (F (2, 20) = 20.38; *p* < 0.0001), 1.0 ± 0.11 vs. 7.9 ± 1.3, *** *p* = 0.0009 (NS vehicle vs. CUS vehicle) and 7.9 ± 1.3 vs. 11 ± 1.4, *p* = 0.2375 (CUS vehicle vs. CUS C2-Crry), n = 7–8 per group. LCN-2, one-way ANOVA (F (2, 21) = 4.930; *p* = 0.0176), 1.0 ± 0.16 vs. 11 ± 4.8, * *p* = 0.0467 (NS vehicle vs. CUS vehicle) and 11 ± 4.8 vs. 12 ± 1.1, *p* = 0.9500 (CUS vehicle vs. CUS C2-Crry), n = 8 per group. VIM, one-way ANOVA (F (2, 21) = 4.692; *p* = 0.0207), 1.0 ± 0.10 vs. 4.2 ± 1.2, * *p* = 0.0223 (NS vehicle vs. CUS vehicle) and 4.2 ± 1.2 vs. 3.5 ± 0.70, *p* = 0.8233 (CUS vehicle vs. CUS C2-Crry), n = 8 per group. *mRNA expression (fold change) of the astrocyte A1 phenotype markers in the PFC:* GATA-1, one-way ANOVA (F (2, 19) = 44.51; *p* < 0.0001), 1.0 ± 0.05 vs. 4.2 ± 0.41, **** *p* < 0.0001 (NS vehicle vs. CUS vehicle) and 4.2 ± 0.41 vs. 1.8 ± 0.13, ^$$$$^ *p* < 0.0001 (CUS vehicle vs. CUS C2-Crry), n = 7–8 per group. H2T23, one-way ANOVA (F (2, 20) = 19.49; *p* < 0.0001), 1.0 ± 0.09 vs. 9.2 ± 1.6, **** *p* < 0.0001 (NS vehicle vs. CUS vehicle) and 9.2 ± 1.6 vs. 4.5 ± 0.27, ^$$^ *p* = 0.0065 (CUS vehicle vs. CUS C2-Crry), n = 7–8 per group. C3, one-way ANOVA (F (2, 21) = 83.61; *p* < 0.0001), 1.0 ± 0.04 vs. 11 ± 0.42, **** *p* < 0.0001 (NS vehicle vs. CUS vehicle) and 11 ± 0.42 vs. 12 ± 1.1, *p* = 0.7535 (CUS vehicle vs. CUS C2-Crry), n = 8 per group. *mRNA expression (fold change) of the astrocyte A2 phenotype markers in the PFC:* CD-109, one-way ANOVA (F (2, 21) = 32.18; *p* < 0.0001), 1.0 ± 0.08 vs. 2.7 ± 0.20, **** *p* = 0.0001 (NS vehicle vs. CUS vehicle) and 2.7 ± 0.20 vs. 3.1 ± 0.26, *p* = 0.4963 (CUS vehicle vs. CUS C2-Crry), n = 8 per group. EMP-1, one-way ANOVA (F (2, 21) = 1.537; *p* = 0.2383), 1.0 ± 0.11 vs. 3.4 ± 1.5, *p* = 0.2533 (NS vehicle vs. CUS vehicle) and 3.4 ± 1.5 vs. 3.0 ± 1.0, *p* = 0.9608 (CUS vehicle vs. CUS C2-Crry), n = 8 per group. S100a, one-way ANOVA (F (2, 21) = 0.008909; *p* = 0.9911), 1.0 ± 0.30 vs. 1.0 ± 0.45, *p* = 0.9982 (NS vehicle vs. CUS vehicle) and 1.0 ± 0.45 vs. 0.96 ± 0.31, *p* = 0.9903 (CUS vehicle vs. CUS C2-Crry), n = 8 per group. (**B**) *mRNA expression (fold change) of the astrocyte activation PAN markers in the hippocampus:* CXCL-10, one-way ANOVA (F (2, 21) = 9.891; *p* = 0.0009), 1.0 ± 0.15 vs. 0.70 ± 0.08 *p* = 0.1293 (NS vehicle vs. CUS vehicle) and 0.70 ± 0.08 vs. 0.34 ± 0.04, *p* = 0.0625 (CUS vehicle vs. CUS C2-Crry), n = 8 per group. LCN-2, one-way ANOVA (F (2, 20) = 1.050; *p* = 0.3683), 1.0 ± 0.75 vs. 1.3 ± 0.50, *p* = 0.8905 (NS vehicle vs. CUS vehicle) and 1.3 ± 0.50 vs. 0.29 ± 0.06, *p* = 0.3558 (CUS vehicle vs. CUS C2-Crry), n = 7–8 per group. VIM, one-way ANOVA (F (2, 21) = 0.09998; *p* = 0.9053), 1.0 ± 1.1 vs. 0.90 ± 0.51, *p* = 0.9940 (NS vehicle vs. CUS vehicle) and 0.90 ± 0.51 vs. 0.58 ± 0.21, *p* = 0.9438 (CUS vehicle vs. CUS C2-Crry), n = 8 per group. *mRNA expression (fold change) of the astrocyte A1 phenotype markers in the hippocampus:* H2T23, one-way ANOVA (F (2, 18) = 32.77; *p* < 0.0001), 1.0 ± 0.13 vs. 1.7 ± 0.23, ** *p* = 0.0052 (NS vehicle vs. CUS vehicle) and 1.7 ± 0.23 vs. 0.17 ± 0.01, ^$$$$^ *p* < 0.0001 (CUS vehicle vs. CUS C2-Crry), n = 6–8 per group. C3, one-way ANOVA (F (2, 20) = 13.23; *p* = 0.0002), 1.0 ± 0.10 vs. 0.84 ± 0.16, *p* = 0.5787 (NS vehicle vs. CUS vehicle) and 0.84 ± 0.16 vs. 0.19 ± 0.01, ^$$^ *p* = 0.0023 (CUS vehicle vs. CUS C2-Crry), n = 7–8 per group. GATA-1, one-way ANOVA (F (2, 12) = 2.132; *p* = 0.1613), 1.0 ± 0.11 vs. 1.6 ± 0.38, *p* = 0.1404 (NS vehicle vs. CUS vehicle) and 1.6 ± 0.38 vs. 1.2 ± 0.09, *p* = 0.4324 (CUS vehicle vs. CUS C2-Crry), n = 4–7 per group. *mRNA expression (fold change) of the astrocyte A2 phenotype markers in the hippocampus:* CD-109, one-way ANOVA (F (2, 18) = 30.55; *p* < 0.0001), 1.0 ± 0.06 vs. 2.6 ± 0.28, **** *p* < 0.0001 (NS vehicle vs. CUS vehicle) and 2.6 ± 0.28 vs. 0.97 ± 0.06, ^$$$$^ *p* < 0.0001 (CUS vehicle vs. CUS C2-Crry), n = 6–8 per group. EMP-1, one-way ANOVA (F (2, 21) = 0.2663; *p* = 0.7688), 1.0 ± 0.86 vs. 1.2 ± 0.42, *p* = 0.9709 (NS vehicle vs. CUS vehicle) and 1.2 ± 0.42 vs. 0.52 ± 0.75, *p* = 0.7573 (CUS vehicle vs. CUS C2-Crry), n = 8 per group. S100a, one-way ANOVA (F (2, 21) = 0.5342; *p* = 0.5939), 1.0 ± 0.25 vs. 0.61 ± 0.09, *p* = 0.6354 (NS vehicle vs. CUS vehicle) and 0.61 ± 0.09 vs. 0.98 ± 0.45, *p* = 0.6642 (CUS vehicle vs. CUS C2-Crry), n = 8 per group. Tukey’s multiple comparison post hoc test was used in the analysis. * *p* < 0.05; ** *p* < 0.01; *** *p* < 0.001 and **** *p* < 0.00001 represent the comparison between NS Vehicle vs CUS Vehicle. ^$$^ *p* < 0.01 and ^$$$$^ *p* < 0.00001 represent the comparison between CUS Vehicle vs CUS C2-Crry.

Similarly to microglia/macrophages, astrocytes also play a key role in brain homeostasis. In addition, activated microglia participate in the transformation of naive astrocytes into reactive astrocytes [45]. A1 neurotoxic reactive astrocytes upregulate genes of the complement pathway implicated in synaptic dysregulation. On the other hand, A2 neuroprotective astrocytes promote neurotrophic factors, which are involved in survival and growth of neurons and synapse repair [45,46,47,48]. We determined the levels of astrocyte reactivity markers, such as “pan”, “A1 neurotoxic”, and “A2 neuroprotective” markers in the PFC and hippocampus. In pan markers, we observed increases in CXCL10, vimentin (Vim), and lipocalin-2 (Lcn2) mRNA levels in the PFC following CUS, suggesting an increased expression of the molecules involved in the trafficking of immune molecules into the PFC following chronic stress (Figure 3A). In A1 neurotoxic markers, CUS exposure increased the levels of C3, GATA-1, and histocompatibility 2, T region locus 23 (H2-T23) in the PFC (Figure 3A). We observed a significant increase in the A2 neuroprotective marker, CD109, while there was no change in the mRNA levels of Epithelial Membrane Protein 1 (EMP1) and S100 calcium-binding protein A (S100a) after exposure to CUS (Figure 3A). C2-Crry treatment significantly attenuated the stress-induced increases in GATA-1 mRNA levels in the PFC, indicating that inhibition of complement activation reduces the stress-induced immune activation in the CNS. In the hippocampus, H2-T23 and CD109 levels were increased following CUS and were attenuated following C2-Crry treatment (Figure 3B). In addition, C2-Crry treatment significantly reduced C3 mRNA levels in mice exposed to CUS. Together, these results indicate that inhibition of complement activation with C2-Crry treatment suppresses the increases in activation of neurotoxic A1 astrocytes after chronic stress.

Overall, the results indicate that targeted inhibition of the complement system using C2-Crry alleviates both inflammation and stress-induced social behavior deficits, highlighting its potential as a therapeutic tool to counteract the adverse effects of chronic stress on the brain.

## 4. Discussion

The focus of this study was to investigate the therapeutic potential of C2-Crry for alleviating chronic-stress-associated behavioral responses and markers of inflammation in the PFC and hippocampus. The complement system was considered as our primary target due to its documented role in driving inflammatory processes associated with numerous neuropsychiatric diseases including anxiety [49], and depression [50,51].

Our initial findings showed a reduction in social behavior deficits induced by chronic stress after the administration of C2-Crry. Earlier studies have established a link between chronic stress and depressive and anxiety-like behavior [52,53] and social behavioral changes [54]. Furthermore, the complement system is associated with negative behavioral stress response in mice [55], with C3 deficiency resulting in decreased systemic IFNβ-induced social behavior deficits [44]. This leads us to hypothesize that C2-Crry attenuates stress-induced social behavioral deficits via mitigation of upstream and/or downstream inflammatory components. Interestingly, C2-Crry is recognized not only for its ability to inhibit complement components, but also for demonstrating disease and behavioral modifying effects in inflammatory conditions [31]. In fact, numerous animal studies have demonstrated that inhibiting the complement system following closed- or open-head brain injuries offers neuroprotection and improves behavioral effects in mice [56,57,58]. These findings have clinical significance, as stress-induced social behavior in mice can resemble the depressive and anhedonic symptoms observed in human subjects with neuropsychiatric disorders [59]. Moreover, chronic stress has been associated with the activation of innate immune system components and symptoms of depression in both rodents and humans [60,61,62].

Our findings also revealed a reduction in chronic stress-induced rises of inflammatory markers in the PFC and hippocampus after treatment with C2-Crry. We found differential effects of chronic stress on inflammatory markers such as iNOS and TNF-α in the PFC and hippocampus. Chronic stress exposure leads to overactivation of the HPA axis, causing prolonged elevations in glucocorticoid levels [63]. This results in structural and functional changes in the brain, particularly in the PFC and hippocampus, regions critical for regulating behavior, emotions, and processing psychological stress [64]. Chronic psychological stress activates brain microglia, which then release significant amounts of pro-inflammatory cytokines such as TNF-α [65]. Microglial activation and corresponding levels of pro-inflammatory cytokines may exhibit notable alterations in the PFC compared to the hippocampus [65]. At the structural level, the PFC is more vulnerable to the effects of chronic stress than the hippocampus [66]. The regional differences in vulnerability to stress may account for the differences observed in the inflammatory markers following chronic stress in our study.

In accordance with our results, there is an established link between chronic stress and the upregulation of inflammatory components. For example, increases of peripheral cytokines have been found in chronic social defeat stress (CSDS) mouse models with subsequent stress-induced behavioral abnormalities [67]. Additionally, chronic stress has also been found to elevate inflammatory complement proteins in the PFC of mice [14] and increase pro-inflammatory TNFα and C-reactive protein (CRP) in rats [68]. Moreover, significant increases in macrophages and IFN-I signaling were found in the PFC and hippocampus of mice subjected to restraint stress [44]. There is evidence indicating that inflammatory conditions lead to greater blood–brain barrier (BBB) dysfunction and permeability in the hippocampus and PFC—regions associated social behavior—permitting the infiltration of peripheral cytokines [69,70]. Furthermore, during increased states of inflammation, C3a/C3aR signaling has been shown to compromise the structure and function of the BBB, leading to poor prognosis and increased neuroinflammation in neurodegenerative disease [71]. Hasantari et al. further explored this by injecting complement inhibitor factor H into the brains of Alzheimer’s disease mouse models, which ultimately resulted in slower disease progression via increased synaptic transmission, reduced complement activation, and reduced cytokine release [72]. These findings suggest that CUS mice may be more vulnerable to disruptions in BBB integrity, resulting in increased infiltration of inflammatory markers into the PFC and hippocampus. Accordingly, our earlier study found an increase in the influx of peripheral monocytes into the PFC following CUS, which was inhibited in C3ar1 KO mice [44]. In the current study, treatment with C2-Crry resulted in attenuation of stress-induced inflammatory markers in the PFC, possibly secondary to the mitigation of complement induced BBB structural and functional changes. These results warrant further investigation into the relationship between targeted complement inhibition and inflammatory changes in brain regions implicated in stress-induced behavioral deficits.

Our findings showed a reduction in the chronic stress-induced elevation of astrocyte activation markers in the PFC and hippocampus after C2-Crry treatment. In this context, prior research has shown stress-induced activation and remodeling of glial cells, specifically microglia/macrophages, in the PFC of mice [73]. Further, Shimoda et al. showed that microglia from mice subjected to restraint stress resulted in higher levels of chemokines and exhibited increased expression of Toll-like 2 receptor (TLR2) mRNA compared to control mice [74]. While extensive research has established a link between stress and glial cell activation in various regions of the brain, such as the PFC and hippocampus [17,75], additional research is needed to develop novel drug targets that may mitigate these stress responses. Specifically, inhibiting complement component C1r results in reduced levels of inflammatory markers, microglia, astrocytes, and degenerating neurons in vivo in Alzheimer’s disease [76]. Moreover, blocking C1q, C3, or the microglial complement receptor CR3 lowers the quantity of phagocytic microglia and mitigates early synapse loss in mouse models of early Alzheimer’s disease [77]. These findings not only underscore the link between glial activation and stress-related disease, but highlight the potential advantages of complement pathway-targeted therapies to mitigate these abnormal inflammatory responses. In addition, our data indicate that CUS induced the expression of damage-associated molecular patterns within the brain, and specifically the subset of phospholipids which have been identified previously as the target of C2-Crry [30].

Our study has a few limitations. First, the current study investigated the potential of systemic administration of C2-Crry on chronic stress-induced inflammatory markers and social behavior. Since complement proteins are also produced in the brain, the effects of complement inhibition in the brain on stress-induced abnormalities in behavior and inflammation are not known. Second, the microglia as well as astrocyte markers were examined in the PFC and hippocampus tissues instead of isolated cells. Third, the effects of complement inhibition on chronic stress-induced alterations in synaptic markers are yet to be determined. Fourth, the effects of complement inhibition on stress susceptibility vs. resilience are not known. Future studies using AAV-mediated complement inhibition in specific brain regions of animal models of chronic stress are warranted to examine the cell-specific role of complement signaling in chronic stress-induced behavior and synaptic changes.

## 5. Conclusions

Targeted inhibition of the complement system using C2-Crry has been shown to alleviate both inflammation and stress-induced social behavior deficits, highlighting its potential as a therapeutic tool to counteract the adverse effects of chronic stress on the brain. These findings hold potential clinical significance, as stress-induced social behavior deficits in mice parallels the depressive and anhedonic symptoms observed in humans with neuropsychiatric disorders like depression, along with the associated heightened inflammatory states that can be linked to depression severity and progression. Therefore, targeted complement inhibition could emerge as a promising, safe, and effective strategy for treating stress-related disorders like anxiety, PTSD, and depression. Future research should evaluate the effectiveness of other targeted complement inhibitors that affect pathways such as CR2-Crry, which are not currently targeted. Importantly, a C3 inhibitor aimed at sites of complement activation is presently undergoing phase 2 clinical trials for anti-neutrophil cytoplasmic autoantibody (ANCA)-associated vasculitis and renal disease [78].

## Data Availability

The original contributions presented in the study are included in the article/Supplementary Material; further inquiries can be directed to the corresponding author.

## Supplementary Materials

The following supporting information can be downloaded at: https://www.mdpi.com/article/10.3390/cells13231988/s1, Table S1. Chronic unpredictable stress paradigm; Table S2. Mouse primers.
