# Supplementary material for: Systemic Administration of a Site-Targeted Complement Inhibitor Attenuates Chronic Stress-Induced Social Behavior Deficits and Neuroinflammation in Mice
Source: Cells. 2024 Dec 2;13(23):1988. doi: 10.3390/cells13231988 (PMC11640647; doi:10.3390/cells13231988)
Supplement: Supplementary file 1 [file cells-13-01988-s001.zip › cells-3287828-supplementary.pdf]

Tables S1. Chronic unpredictable stress paradigm

	Day 1	Day 2	Day 3	Day 4	Day 5	Day 6	Day 7
**Week 1**	***Morning:*** Confinement (1 hr)<br><br>***Mid-day:*** Forced Swim (30 min)<br><br>***Evening:*** Paired Housing (10 min)	***Morning:*** Empty water bottle (2 hrs)<br><br>***Mid-day:*** Confinement (1 hr)	***Morning:*** Forced swim (30 min)<br><br>***Evening:*** Food Restriction (15 hrs)	***Morning:*** Inversion of light/dark (1 hr)<br><br>***Evening:*** Paired Housing (10 min)	***Evening:*** Empty water bottle (2 hrs)	***Morning:*** Paired Housing (10 min)	***Mid-day:*** Inversion of light/dark (1 hr)
	**Day 8**	**Day 9**	**Day 10**	**Day 11**	**Day 12**	**Day 13**	**Day 14**
**Week 2**	***Morning:*** Forced swim (30 min)<br><br>***Mid-day:*** Confinement (1 hr)<br><br>***Evening:*** Food Restriction (15 hrs)	***Morning:*** Confinement (1 hr)<br><br>***Mid-day:*** Paired Housing (10 min)	***Morning:*** Empty water bottle (2 hrs)<br><br>***Evening:*** Inversion of light/dark (1 hr)	***Morning:*** Paired Housing (10 min)<br><br>***Mid-day:*** Forced Swim (30 min)<br><br>***Evening:*** Confinement (1 hr)	***Mid-day:*** Empty water bottle (2 hrs)	***Mid-day:*** Inversion of light/dark (1 hr)	***Mid-day:*** Paired Housing (10 min)
	**Day 15**	**Day 16**	**Day 17**	**Day 18**	**Day 19**	**Day 20**	**Day 21**
**Week 3**	***Morning:*** Confinement (1 hr)<br><br>***Mid-day:*** Forced Swim (30 min)<br><br>***Evening:*** Paired Housing (10 min)	***Morning:*** Empty water bottle (2 hrs)<br><br>***Mid-day:*** Confinement (1 hr)	***Morning:*** Forced swim (30 min)<br><br>***Evening:*** Food Restriction (15 hrs)	***Morning:*** Inversion of light/dark (1 hr)<br><br>***Evening:*** Paired Housing (10 min)	***Evening:*** Empty water bottle (2 hrs)	***Mid-day:*** Paired Housing (10 min)	***Mid-day:*** Inversion of light/dark (1 hr)

Tables S2. Mouse primers

Gene	Forward Primer	Reverse Primer
iNOS	CCC TTC AAT GGT TGG TAC ATG G	ACA TTG ATC TCC GTG ACA GCC
TNF-α	CGT CAG CCG ATT TGC TAT CT	CGG ACT CCG CAA AGT CTA AG
IL-1β	TGT AAT GAA AGA CGG CAC ACC	TCT TCT TTG GGT ATT GCT TGG
CD-32	CTG GAA GAA GCT GCC AAA AC	CCA ATG CCA AGG GAG ACT AA
CD-86	GAG CGG GAT AGT AAC GCT GA	GGC TCT CAC TGC CTT CAC TC
SOCS-3	CGT TGA CAG TCT TCC GAC AA	TAT TCT GGG GGC GAG AAG AT
ARG-1	GTG AAG AAC CCA CGG TCT GT	GCC AGA GAT GCT TCC AAC TG
IL-10	GGC TGA GGC GCT GCT ATC G	TCA TTC ATG GCC TTG TAG ACA CC
TGF-β	CTT TTG ACG TCA CTG GAG TTG	CAG TGA GCG CTG AAT CGA A
B2M	CCC CAC TGA GAC TGA TAC ATA CG	CGA TCC CAG TAG ACG GTC TTG
CXCL-10	GCC GTC ATT TTC TGC CTC AT	GCT TCC CTA TGG CCC TCA TT
LCN-2	GGA CCA GGG CTG TCG CTA CT	GGT GGC CAC TTG CAC ATT GT
VIM	CGG AAA GTG GAA TCC TTG CAG G	AGC AGT GAG GTC AGG CTT GGA A
C3	AGC TTC AGG GTC CCA GCT AC	GCT GGA ATC TTG ATG GAG ACG C
GATA-1	ACC GCC CGG ATG TTT TGA C	TGA CGT AAA ATA TGA CCC GAT GG
H2-T23	GGA CCG CGA ATG ACA TAG C	GCA CCT CAG GGT GAC TTC AT
CD-109	CAC AGT CGG GAG CCC TAA AG	GCA GCG ATT TCG ATG TCC AC
EMP-1	GAG ACA CTG GCC AGA AAA GC	TAA AAG GCA AGG GAA TGC AC
S100α	CCT CTG GCT GTG GAC AAA AT	CTG CTC ACA AGA AGC AGT GG
